# RETRACTION: Antiproliferative Effect of Rottlerin on Sk‐Mel‐28 Melanoma Cells

**DOI:** 10.1155/ecam/9832583

**Published:** 2026-04-07

**Authors:** Evidence-Based Complementary and Alternative Medicine

RETRACTION: E. Daveri, G. Valacchi, R. Romagnoli, E. Maellaro, and E. Maioli, “Antiproliferative Effect of Rottlerin on Sk‐Mel‐28 Melanoma Cells,” *Evidence-Based Complementary and Alternative Medicine*, 2015, 545838, https://doi.org/10.1155/2015/545838.

The above article, published online on 28 May 2015 in Wiley Online Library (https://wileyonlinelibrary.com), has been retracted by John Wiley & Sons Ltd. The retraction has been agreed due to concerns raised with the figures. Specifically:

• After resizing, the ß‐actin bands in Figure 2 appear highly similar to lanes 2–5 of the first set of ß‐actin bands shown in Figure 2 of [1].
• In Figure 4, there are areas of the Western blot background that appear identical between lanes 2 and 4 of Cyclin D1 and lanes 1 and 3 of p21/Cip1
• Multiple other blots in the article show evidence of undeclared splicing

The authors provided a response to the concerns but were unable to provide the original western blots. After assessment, the results and conclusions presented can no longer be considered reliable, and the article is retracted.

The authors disagree with the retraction.
